# Calcification Patterns in Papillary Thyroid Carcinoma are Associated with Changes in Thyroid Hormones and Coronary Artery Calcification

**DOI:** 10.3390/jcm7080183

**Published:** 2018-07-26

**Authors:** Jeonghoon Ha, Jeongmin Lee, Kwanhoon Jo, Jeong-Sun Han, Min-Hee Kim, Chan Kwon Jung, Moo IL Kang, Bong Yeon Cha, Dong-Jun Lim

**Affiliations:** 1 Division of Endocrinology and Metabolism, Department of Internal Medicine, College of Medicine, The Catholic University of Korea, Seoul 06591, Korea; hajhoon@catholic.ac.kr (J.H.); 082mdk45@gmail.com (J.L.); lovi@naver.com (K.J.); winehan@me.com (J.-S.H.); benedict@catholic.ac.kr (M.-H.K.); mikang@catholic.ac.kr (M.I.K.); bycha@catholic.ac.kr (B.Y.C.); 2 Department of Hospital Pathology, College of Medicine, The Catholic University of Korea, Seoul 06591, Korea; ckjung@catholic.ac.kr

**Keywords:** calcification, papillary thyroid carcinoma, thyroid hormones, coronary artery calcification, SPINA-GT, TSH index

## Abstract

Recent studies suggested that a lower serum thyroid hormone level is associated with more vascular calcification. However, it has been rarely evaluated whether lower thyroid hormone levels affect the calcification of thyroid cancer and there is a relationship between calcification patterns of papillary thyroid carcinoma (PTC) and coronary artery calcification (CAC). The study was divided into two groups: First, we retrospectively reviewed 182 PTC patients and examined the correlation between PTC calcification patterns and CAC by coronary computed tomography (CT). Second, the correlation between the calcification pattern of PTC and thyroid hormone concentration was investigated (*n* = 354). The calcification pattern of PTC was evaluated by thyroid ultrasonography and classified into four groups: no-calcification, microcalcification, macrocalcification, and mixed-calcification. In PTC patients with microcalcification and mixed calcification, more CAC was observed and coronary calcium score (CCS) was higher. Lower free T4 and higher thyroid-stimulating hormone (TSH) levels were associated with microcalcification and mixed calcification, not with macrocalcification and no calcification. PTC with microcalcification and mixed calcification showed more aggressive phenotypes like lymph node metastasis and more advanced TNM (tumor, node, and metastasis) stage than those with no calcification and macrocalcification. Calcification patterns of PTC showed close association with thyroid hormone levels and CAC. Further research is needed to determine how these findings are related to cardiovascular risk and disease-specific mortality.

## 1. Introduction

Thyroid cancer is the most common endocrine tumor, and its incidence has increased over the past two decades. Papillary thyroid carcinoma (PTC) is the most common type of thyroid cancer and frequently shows microcalcification on ultrasonography (US), which is the most remarkable finding indicating PTC. Microscopically, microcalcification observed in PTC is termed psammoma bodies, and the mechanism for their formation is not clear [1]. Macrocalcification on US is observed mainly in benign nodules, especially in older age groups, and is occasionally observed in thyroid cancer [2]. Several other types of thyroid calcification have been reported, but the clinical implications are still unclear [3].

Recent studies suggested that lower serum thyroid hormone level is associated with more vascular calcification [4,5,6]. Coronary artery calcification (CAC) is associated with an increased risk of cardiovascular morbidity and mortality [7,8]. The coronary calcium score (CCS), which can be measured with coronary computed tomography (CT), provides information about the presence and extent of calcified plaques in coronary arteries and is a strong and independent predictor of cardiovascular disease (CVD) risk [9]. In breast cancer, breast artery calcification is associated with a higher CCS and increased CVD risk [10,11]. However, it has been unknown whether intratumoral calcification per se within thyroid cancer is associated with vascular calcification. Herein, we investigated CAC and CCS in patients with PTC, according to intratumoral calcification patterns. We also examined how these findings are related to thyroid hormones.

## 2. Material and Methods

### 2.1. Study Group

#### 2.1.1. Group for Observing the Association of Calcification in PTC and Coronary Artery Calcification (Group 1)

We retrospectively analyzed the records of 210 patients that underwent thyroidectomy for thyroid malignancy and coronary CT at our institution from October 2008 to April 2017. We excluded patients taking anti-thyroid medications or thyroid hormones at the time of surgery (*n* = 5), with insufficient pre- or postoperative laboratory results (*n* = 18), whose pathology was confirmed as another type of thyroid carcinoma (*n* = 4) or as a benign nodule after surgery (*n* = 1). Finally, 182 patients were included in this analysis (Supplementary Materials Figure S1A). Of 182 patients who underwent coronary CT, 93 patients underwent coronary CT as a health screening without definite symptoms or signs of coronary artery disease and 78 patients underwent for the evaluation of cause of symptoms such as exertional dyspnea or intermittent chest pain. In the remaining 11 patients, no definite cause of coronary CT could be identified. The presence of CAC was confirmed in all 182 subjects, but CCS was measured with the same protocol by radiologist only in 112 subjects. This study was approved by the institutional review board (KC17RESI0268).

#### 2.1.2. Group for Observing the Association of Calcification Pattern in PTC According to Thyroid Hormones (Group 2)

We retrospectively reviewed the records of patients who had undergone thyroidectomy for thyroid malignancy at tertiary referral hospital, Seoul, Korea, between January 2013 and December 2013. Among 396 patients, we excluded those taking antithyroid medications or thyroid hormones at the time of surgery (*n* = 4), with no preoperative thyroid US imaging (*n* = 12), with insufficient pre- or postoperative laboratory results (*n* = 18), whose pathology was confirmed as medullary thyroid carcinoma (*n* = 4), and whose nodules measured <3 mm (*n* = 4). A total of 42 patients were excluded and the records of 354 patients were included in the analysis (Supplementary Materials Figure S1B). The patient groups did not overlap, since no subject was included in both groups. This study was also approved by the institutional review board.

### 2.2. Measurements

The serum concentrations of FT4, T3, and thyroid-stimulating hormone (TSH) were measured by enzyme immunoassay using a commercially IRMA kit (Beckman Coulter, Brea, CA, USA). The reference ranges of FT4, T3, and TSH were 0.82–1.76 ng/dL, 0.6–1.81 ng/mL, and 0.55–4.78 μIU/mL, respectively. Thyroid US was performed in our hospital by a surgeon, radiologist, or endocrinologist before the surgery. The presence of calcification was documented by a physician who performed an US examination of the thyroid and was confirmed by one of our researchers. Cohen’s κ value was calculated to confirm the inter-observer variability. In most instances, we performed neck CT before surgery to confirm the presence of metastasis to the surrounding lymph nodes. In addition to thyroid US, we refer to neck CT findings to confirm the calcification pattern, which was mainly macrocalcification. The presence of vascular calcification was identified by coronary CT, and the extent of calcification was confirmed by CCS when reviewing the medical records retrospectively. To determine the secretory capacity of the thyroid gland, SPINA-GT is assessed using the formula presented in previous studies by Dietrich et al. [12,13,14,15]. The reference range is between 1.4 and 8.7 pmol/s [16]. Jostel’s TSH index (TSHI) as a quantitative marker for pituitary thyrotropic function is calculated as TSHI = log TSH + 0.1345 × FT4 [17]. The standardized TSHI (sTSHI) is calculated using a z-transformed value incorporating mean (2.7) and standard deviation (0.676) of TSHI in a reference population [15]. Its reference range is suggested to be between −2 and +2 [15]. In the present study, sTSHI was analyzed.

### 2.3. Calcification Pattern in Thyroid Cancer

In the present study, the calcification pattern was classified into the following four groups according to the consensus statement and recommendations proposed by the Korean Society of Thyroid Radiology and the Korean Society of Radiology [18]: (1) No calcification: no calcification was confirmed by both US and neck CT; (2) Microcalcification: presence of punctuate echogenic foci of ≤1 mm either with or without posterior shadowing, which may not always be visible in neck CT; (3) Macrocalcification: presence of echogenic foci >1 mm with posterior shadowing and confirmed by neck CT; (4) Mixed calcification (mix of microcalcification and macrocalcification): presence of mixed features of microcalcification and macrocalcification confirmed by US and neck CT.

**Figure 1:**
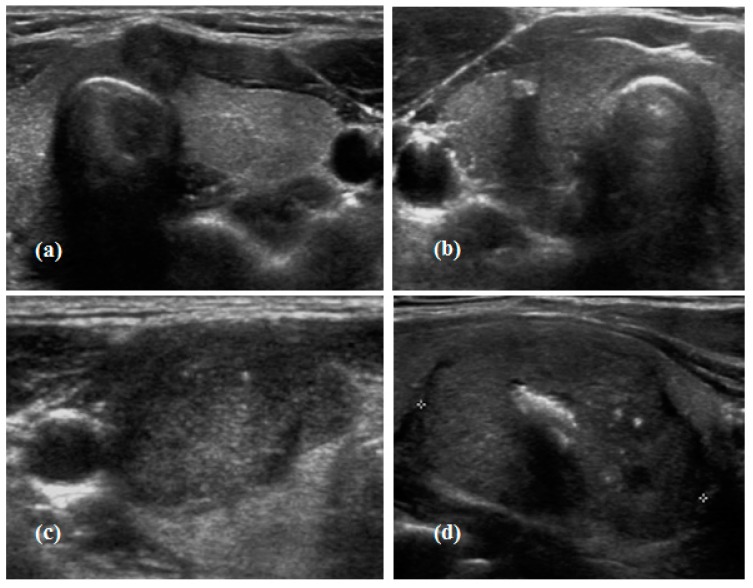
Transverse ultrasound imaging of calcification patterns observed in the study groups according to the extent of calcification. (**a**) No calcification; (**b**) macrocalcification, echogenic foci >1 mm with posterior shadowing; (**c**) microcalcification, punctuated echogenic foci of <1 mm without posterior shadowing, brighter echo than the surrounding thyroid tissue; (**d**) mixed calcification, a combination of macrocalcification and microcalcification.

There is no clear definition of mixed-calcification in the consensus statement mentioned above. In this study, mixed calcification was defined as the combination of microcalcification type and macrocalcification type. Figure 1 shows representative cases of thyroid US imaging identified in our patients.

Interobserver variability was calculated using the measurements taken by four physicians who specialize in thyroid US and was calculated using Cohen’s κ. The κ values for each US feature were: 0.875 (no calcification), 0.877 (macrocalcification), 0.777 (microcalcification), and 0.725 (mixed calcification).

### 2.4. Definition of Metabolic Syndrome and Criteria for Diagnosis

To confirm the metabolic status, the presence of metabolic syndrome and its components used for diagnosis were identified. Metabolic syndrome was defined in accordance with the American Heart Association/National Heart, Lung and Blood Institute criteria [19], including a modification in the waist circumference (WC) value according to the World Health Organization—Asian Pacific region criteria for abdominal obesity. This entity was defined if three or more of the criteria were present, as follows: (1) WC ≥ 90 cm for men or ≥80 cm for women; (2) fasting TG ≥ 150 mg/dL or anti-hyperlipidemic medication use; (3) high-density lipoprotein-cholesterol <40 mg/dL in men or <50 mg/dL in women or anti-hyperlipidemic medication use; (4) elevated blood pressure (systolic blood pressure/diastolic blood pressure ≥130/85 mmHg) or daily use of antihypertensive medication; (5) fasting glucose ≥100 mg/dL or current use of insulin or oral hypoglycemia medication.

### 2.5. Statistical Analysis

Statistical analyses were performed using SAS (version 9.1; SAS Institute, Cary, NC, USA). Data are expressed as mean ± SD or percentage unless otherwise stated. Multiple comparisons were made by analysis of variance (ANOVA), and Tukey’s honestly significance difference if appropriate. Pearson’s chi-square test was used to determine a relationship between categorical variables. To identify independent associations between thyroid function and calcification patterns of cancer as a continuous measure, multiple linear regression analysis was performed. A subgroup analysis stratified by calcification groups was done using analysis of covariance. *p*-Values < 0.05 were considered significant. To evaluate the inter-observer agreement for the calcification patterns observed in US, Cohen’s κ value was calculated. The interobserver or intraobserver agreement was classified as no (0.01–0.20), fair (0.21–0.40), moderate (0.41–0.60), substantial (0.61–0.80), and almost perfect (0.81–1.00) agreement [20].

## 3. Results

### 3.1. Relationship between Calcification Patterns of PTC and CAC

The 182 patients in group 1 were divided into four groups based on the calcification patterns, and their coronary calcification status was analyzed (Table 1).

**Figure 2:**
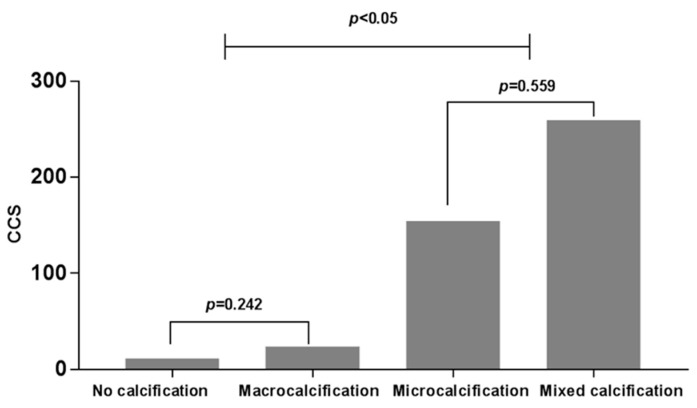
Coronary calcium score (CCS) according to PTC calcification pattern measured in 112 of 182 subjects. *p*-value by one-way ANOVA.

Individuals with the mixed-calcification were oldest and most of this group was female. CAC evaluated by coronary CT was observed most frequently in the mixed-calcification group and least frequently in the no-calcification group. The CCS was confirmed in 112 patients out of 182 patients who underwent coronary CT. The CCS was higher in the microcalcification and mixed calcification groups than in the no calcification and macrocalcification groups (*p* = 0.000) (Table 1 and Figure 2).

In the mixed-calcification group, BMI was 26.85 kg/m^2^ and the prevalence of metabolic syndrome was 71%. However, information about waist circumference required for the diagnosis of metabolic syndrome was missing in this analysis, and the prevalence of metabolic syndrome may have been higher if this information had been available and included.

### 3.2. Relationships between Calcification Patterns of PTC and Thyroid Hormone Concentrations

Among the 354 patients with PTC, the mean age was 46 years, and 275 (77.7%) were women. No calcification was confirmed in 162 (45.8%) patients, macrocalcification in 42 (11.9%), microcalcification in 108 (30.5%), and mixed calcification in 42 (11.9%). At the time of surgery, lymph node metastasis was confirmed in 167 patients (47.2%) (Table 2).

The patients were divided into four groups according to the four calcification patterns, and the relationships between the calcification patterns and clinical parameters were assessed (Table 3).

Age was significantly higher in the macrocalcification group and the mixed-calcification group (*p* = 0.000). The tumor size was also larger in these two groups (1.31 ± 0.94 and 1.27 ± 0.77 cm), respectively (*p* = 0.000). The prevalence rates of lymph node metastasis and metastatic lymph node number were higher in the microcalcification and mixed calcification groups than in the no calcification and macrocalcification groups (Table 3). The lymph node ratio was defined as the number of metastatic lymph nodes divided by the number of lymph nodes removed. This ratio was higher in the microcalcification and mixed calcification groups, but no statistical difference between groups was observed (*p* = 0.103). A higher percentage of patients with microcalcification or mixed calcification had a higher stage of disease (stage III or IV). FT4 concentration decreased gradually from the no calcification group to the mixed calcification group (*p* = 0.000); TSH concentration showed an increasing trend (*p* = 0.032) (Table 3). There was no statistically significant difference in SPINA-GT in four groups, but it was found to be low in the mixed calcification group (Table 3).

### 3.3. Free T4 and TSH Concentrations According to the Four Calcification Patterns

For free T4 and TSH levels, both the crude values and values adjusted for factors that might affect FT4 concentration were included in the analyses (Table 4).

Both the crude and adjusted FT4 concentrations decreased gradually from the no-calcification to the mixed calcification groups (*p* = 0.000 and *p* < 0.042, respectively). TSH concentration was higher in microcalcification and mixed calcification groups in both models (*p* = 0.032 and *p* = 0.004, respectively) (Table 4). FT4 concentrations did not differ significantly between the no-calcification and macrocalcification groups, or between the microcalcification and mixed calcification groups.

## 4. Discussion

CAC was observed in 57% and 64% of the microcalcification and mixed calcification groups of PTC patients, respectively, compared to 17% in the no-calcification group and CCS was higher in these two groups. A high CCS is associated with a greater risk of cardiovascular events [21,22]. Based on the previous analysis of the association between FT4 level and vascular calcification [4,23], we sought to determine the relationship between vascular calcification and PTC intratumoral calcification, and to investigate the relationship between PTC calcification and thyroid hormones. Our hypothesis is presented in Supplementary Materials Figure S2. Microcalcification and mixed-calcification types of PTC were associated with more CAC, and these specific patterns of PTC calcification were also related to lower FT4 and higher TSH concentrations.

There have been cross-sectional, population-based studies on the association of thyroid hormones with vascular calcification. However, the results are not consistent depending on the type of thyroid hormone. In general, studies have shown consistent results on the effect of lower FT4 on vascular calcification or metabolic parameters, but the effect of TSH has not been concordant yet. Low-normal FT4 level is associated with various metabolic parameters related to atherosclerosis or atherosclerotic vascular changes [24,25,26] and is significantly related to insulin resistance and metabolic syndrome, which in turn increase CVD risk [27,28,29]. Previously, we conducted a cross-sectional study of 669 healthy adults and found that FT4, but not TSH correlated inversely with coronary artery calcification [6]. A recent retrospective study of 2173 patients by another study group found that low baseline FT4 level was associated with a high risk of coronary artery calcium score progression over four years [4]. The biologic mechanisms underlying this was not clear, but some researchers have suggested that the serum FT4 level could be a more sensitive indicator of cardiac thyroid status than serum TSH level [4,30]. 

Based on previous findings on the association of thyroid hormones with vascular calcification, we investigated the relationship between thyroid hormones and PTC calcification. In our study, the concentration of thyroid hormones varied according to the calcification patterns of PTC. It was found that FT4 concentration was highest in no calcification group and lowest in the microcalcification and mixed calcification groups. In the contrary, TSH was the lowest in no calcification group and was higher in microcalcification and mixed calcification groups. This difference was significant even after adjusting for other variables that might affect FT4 and TSH concentration. In addition, BMI and the percentage of patients with metabolic syndrome were higher in the mixed calcification group than in the other groups. These changes in metabolic components are similar to those observed in subjects with overt hypothyroidism [6,27,31].

The mechanism by which the association of thyroid hormone with PTC calcification is not yet clear. Recent studies have suggested that molecules such as thyroid hormone receptor beta (THRβ) and Runt Related Transcription Factor 2 (RUNX2) may be involved in the mechanism responsible for calcification in thyroid cancer [32]. The role of RUNX2 in osteoblastic differentiation and calcification has been demonstrated in previous studies [33,34], and recently Carr et al. suggested that dysregulation of THRβ, which acts as a tumor suppressor gene, and the concomitant increase in the expression of RUNX2, which acts as a tumor promotor, are associated with the development of thyroid cancer [32]. Therefore, lower thyroid hormone levels, which may further allow decreased signal to THRβ, might induce cancer-specific RUNX2 overexpression and finally present microcalcification specific to PTC, and mixed calcification, which consists of accumulated and combined microcalcifications. It could be speculated that lower thyroid hormone levels interconnect PTC calcification and vascular calcification (Supplementary Materials Figure S2).

Interestingly, the prevalence of each calcification pattern was similar to that of previous studies. A previous study showed the 46%, 35%, and 15% of PTC patients show no calcification, microcalcification, and macrocalcification, respectively [35]. These values are similar to the respective percentages of 45.8% 30.5% and 11.9% observed in our study. Microcalcification observed in solid hypoechoic nodules is thought to be highly suggestive of malignancy, with a reported specificity of 84–97% [18,36,37], and its presence is associated with poorer disease-free survival and prognosis [38]. The characteristics of the macrocalcification and mixed calcification groups are interesting. The macrocalcification group, which accounted for 11.9% of the patients in this study, was the oldest (54.6 years) and had the largest tumors (1.31 cm). Lymph node metastasis was less frequently observed, and more patients had TNM stage I or II than stage III and IV. Our results seem to be consistent with previous studies showing that isolated macrocalcification in PTC is related to older age [38]. Another unique result relates to the characteristics of the mixed calcification group. Mixed calcification is defined as the presence of both microcalcification and macrocalcification. It is not merely a morphological mixture but also has characteristics of both microcalcification and macrocalcification. Clinical variables in the mixed calcification group, such as age and tumor size, resembled those in the macrocalcification group, whereas variables related to the prognosis were more similar to those in the microcalcification group. The lymph node ratio was 16.4% in the mixed-calcification group, which was the next highest after the microcalcification group (17.1%). The FT4 concentration was the lowest among the four groups.

The occurrence of mixed calcification may be explained by the following possible mechanisms. First, the production of macrocalcification reflects the combination of numerous microcalcifications. Some of the microcalcifications may merge into larger macrocalcification and some may remain in the form of microcalcification, which ultimately retains the characteristic of microcalcifications. A second possible mechanism may be that the macrocalcification observed in mixed calcification may occur independently of microcalcification. Studies have shown that macrocalcification is associated with older age in both benign nodules [2] and PTC [38]; therefore, the occurrence of macrocalcification may reflect a natural course in thyroid nodules. 

A limitation of this study is that our findings do not imply a causal relationship because of the retrospective study design and involvement of two different study groups. In addition, it is necessary to confirm parameters such as serum calcium, serum parathyroid hormone, and serum 25-hydroxyvitamin D involved in the development of calcification. Despite the limitations, this study confirmed the mutual relevance of thyroid hormones, PTC calcification, and CAC, which will provide a good basis for further research. 

## 5. Conclusions

To our knowledge, this is the first study to identify the association between PTC calcification, CAC, and thyroid hormones. The microcalcification and mixed calcification groups of PTC patients were found to be highly related to CAC. We also found lower FT4 and higher TSH concentration in these groups. Further investigation is needed to determine whether PTC patients with microcalcification or mixed calcification are associated with CVD events and have potentials to increase disease-specific mortality or to decrease disease-free survival.

## Figures and Tables

**Table 1:** Relationship between four papillary thyroid carcinoma (PTC) calcification patterns and coronary artery calcification / metabolic parameters (Group 1).

	No Calcification (*n* = 59)	Macrocalcification (*n* = 26)	Microcalcification (*n* = 69)	Mixed Calcification (*n* = 28)	*p*-Value
Age	61.39 ± 8.39	65.92 ± 9.36	63.67 ± 10.14	66.64 ± 12.93	0.080 *
Female (%)	48 (81%)	18 (69%)	39 (57%)	17 (61%)	0.023
Coronary calcification ^a^ (%)	10 (17%)	15 (58%)	39 (57%)	18 (64%)	0.000
CCS ^b^	11.39 ± 25.03	23.76 ± 40.27	154.39 ± 490.79	259.48 ± 737.90	0.179 *
BMI (kg/m^2^)	25.38 ± 3.48	25.37 ± 3.64	24.86 ± 2.61 ^‡^	26.85 ± 4.18 ^†^	0.072 *
Number of components satisfying the requirements for MetS diagnosis ^c^					0.080
0	4	1	7	0	
1	5	4	8	0	
2	19	7	18	5	
3	12	8	8	8	
4	14	3	20	12	
MetS ^d^	26 (44%)	11 (42%)	28 (41%)	20 (71%)	0.026

^a^ Coronary calcification was confirmed by coronary computed tomography; ^b^ CCS, coronary calcium score, estimation ± SE; CCS was measured in 112 of 182 patients; ^c^ The criteria for diagnosis of metabolic syndrome (MetS) were according to the National Cholesterol Education Program (NCEP) Adult Treatment Panel III diagnostic criteria; ^d^ In patients who displayed three or more components and who were diagnosed as having MetS, the diagnosis of MetS may have been underestimated because the waist circumference was not identified. In the analyses, CCS, fasting glucose concentration, and concentrations of triglyceride and high-density lipoprotein cholesterol, and systolic and diastolic blood pressure were adjusted for age and sex, BMI, body mass index; MetS, metabolic syndrome; * *p*-value by one-way ANOVA. ^†^ *p* < 0.05 compared with the microcalcification group by one-way ANOVA and post-hoc analysis; ^‡^ *p* < 0.05 compared with the mixed calcification group by one-way ANOVA and post-hoc analysis; Tukey’s honest significance difference test was applied for post-hoc analysis.

**Table 2:** Association of calcification pattern in PTC according to thyroid hormones (Group 2).

Clinical Parameters	Number (*n* = 354)
Age (years)	46.08 ± 12.22
Female (%)	275 (77.7%)
Tumor size (cm)	0.99 ± 0.87
Patterns of calcification ^a^	
No calcification (%)	162 (45.8%)
Macrocalcification (%)	42 (11.9%)
Microcalcification (%)	108 (30.5%)
Mixed calcification ^b^ (%)	42 (11.9%)
Extrathyroidal extension (%)	141 (39.8%)
Lymph node metastasis (%)	167 (47.2%)
TNM stage ^c^ (%)	
I	142 (40.1%)
II	11 (3.1%)
III	154 (43.5%)
IV	46 (13.0%)
Free T4 (ng/dL)	1.33 ± 0.20
T3 (ng/mL)	1.11 ± 0.40
TSH (μIU/mL)	2.61 ± 2.12
Anti-TPO Ab (U/L)	106.33 ± 268.49

^a^ Adapted classification suggested by Korean Society of Thyroid Radiology and Korean Society of Radiology; ^b^ mixed type refers to the combination of microcalcification and macrocalcification; ^c^ TNM (tumor, node, and metastasis) staging was based on the American Joint Committee on Cancer–TNM 7th edition; data expressed as mean ± standard deviation. TSH, thyroid-stimulating hormone; TPO, Thyroperoxidase.

**Table 3:** Clinical differences between the four calcification patterns (Group 2).

	No Calcification (*n* = 162)	Macrocalcification (*n* = 42)	Microcalcification (*n* = 108)	Mixed Calcification (*n* = 42)	*p*-Value
Age (years)	44.27 ± 10.68 ^‡^	54.60 ± 12.24 ^†,^^§^	44.17 ± 13.09 ^‡^	49.48 ± 11.55	0.000 *
Female (%)	127 (78.4%)	36 (85.7%)	80 (74.1%)	32 (76.2%)	0.480
Tumor size (cm)	0.78 ± 0.85 ^‡,^^§,^^ǁ^	1.31 ± 0.94 ^†^	1.07 ± 0.83	1.27 ± 0.77 ^†^	0.000 *
Extrathyroidal extension (%)	48 (29.6%)	23 (54.8%)	45 (41.7%)	25 (59.5%)	0.000
Lymph node metastasis (%)	58 (35.8%)	18 (42.9%)	65 (60.2%)	26 (61.9%)	0.000
Number of LN metastasis	1.53 ± 3.74 ^§,^^ǁ^	3.17 ± 6.28	3.69 ± 6.99 ^†^	4.17 ± 5.76 ^†^	0.003 *
LNR (%)	11.04 ± 20.13	14.38 ± 20.84	17.12 ± 22.45	16.40 ± 18.22	0.103
TNM stage (%)					0.000
I	85 (52.8%)	14 (33.3%)	32 (29.6%)	11 (26.2%)	
II	3 (1.2%)	3 (7.1%)	5 (4.6%)	1 (2.4%)	
III	65 (40.4%)	19 (45.2%)	51 (47.2%)	19 (45.2%)	
IV	9 (5.6%)	6 (14.3%)	20 (18.5%)	11 (26.2%)	
Free T4 (ng/dL)	1.39 ± 0.18 ^§,^^ǁ^	1.36 ± 0.23 ^ǁ^	1.28 ± 0.17 ^†^	1.22 ± 0.22 ^†,‡^	0.000 *
T3 (ng/mL)	1.08 ± 0.18	1.06 ± 0.15	1.16 ± 0.68	1.13 ± 0.21	0.360 *
TSH (μIU/mL)	2.37 ± 1.37 ^ǁ^	2.26 ± 1.88	2.80 ± 2.63	3.31 ± 2.95 ^†^	0.032 *
Anti-TPO Ab (U/L)	118.86 ± 300.33	97.10 ± 216.68	99.79 ± 259.17	84.03 ± 268.49	0.861 *
SPINA-GT (pmol/s) ^a^	4.40 ± 6.14	3.70 ± 1.72	3.79 ± 6.39	2.77 ± 1.08	0.368 *
sTSHI ^b^	0.55 ± 1.12	0.35 ± 1.10	0.44 ± 1.09	0.50 ± 1.21	0.741 *

LN, Lymph node; LNR, Lymph node ratio. ^a^ Reference range for SPINA-GT: 1.4–8.7 pmol/s; ^b^ sTSHI, standardized TSH index, reference range: −2–+2; * *p*-value by one-way ANOVA; ^†^ *p* < 0.05 compared with No calcification group by one-way ANOVA and post-hoc analysis; ^‡^ *p* < 0.05 compared with macrocalcification group by one-way ANOVA and post-hoc analysis; ^§^ *p* < 0.05 compared with microcalcification group by one-way ANOVA and post-hoc analysis; ^ǁ^ *p* < 0.05 compared with mixed calcification group by one-way ANOVA and post-hoc analysis; Tukey’s honest significance difference test was applied for post-hoc analysis; data are expressed as means ± standard deviation.

**Table 4:** Crude and adjusted mean free T4 and TSH concentrations according to the four calcification patterns.

	Free T4 (ng/dL)		TSH (μIU/mL)	
	Crude	Adjusted ^a^	Crude	Adjusted ^b^
No calcification	1.39 ± 0.02	1.42 ± 0.02	2.37 ± 0.17	2.34 ± 0.21
Macrocalcification	1.36 ± 0.03	1.43 ± 0.04	2.26 ± 0.32	2.40 ± 0.46
Microcalcification	1.28 ± 0.02	1.29 ± 0.02	2.81 ± 0.20	2.82 ± 0.23
Mixed calcification	1.22 ± 0.03	1.23 ± 0.03	3.31 ± 0.32	3.37 ± 0.38
*p*-value	0.000	0.042	0.032	0.004

^a^ Adjusted for sex, age, tumor size, and serum TSH concentration (estimation ± standard error); ^b^ Adjusted for sex, age, size, serum free T4 and T3 concentrations, and anti-TPO antibody titer; data are expressed as estimation ± standard error.

## Supplementary Materials

The following are available online at http://www.mdpi.com/2077-0383/7/8/183/s1, Figure S1: Flow chart for study population enrollment. (A) Group for observing the association of calcification in PTC and coronary artery calcification (Group 1), (B) Group for observing the association of calcification pattern in PTC according to thyroid hormones (Group 2), Figure S2: A schematic diagram of the study hypothesis. The solid lines indicate knowledge confirmed by several references, and the dotted lines indicate the hypothesis tested in this study. The mechanism for the relationships has not been clarified, even for information confirmed by published references.
