# Supplementary material for: Calcification Patterns in Papillary Thyroid Carcinoma are Associated with Changes in Thyroid Hormones and Coronary Artery Calcification
Source: J Clin Med. 2018 Jul 26;7(8):183. doi: 10.3390/jcm7080183 (PMC6111282; doi:10.3390/jcm7080183)
Supplement: Supplementary file 1 [file jcm-07-00183-s001.pdf]

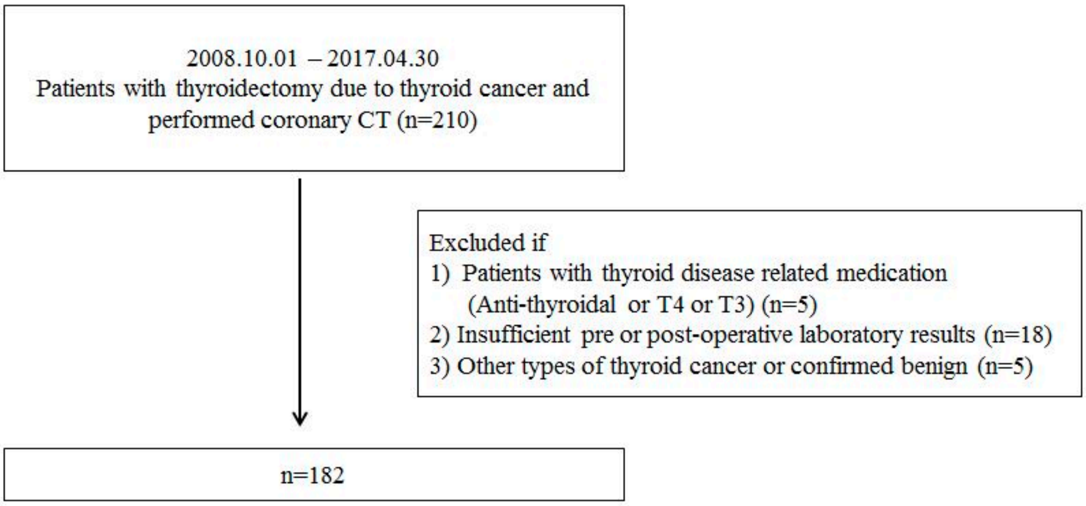

(**a**)

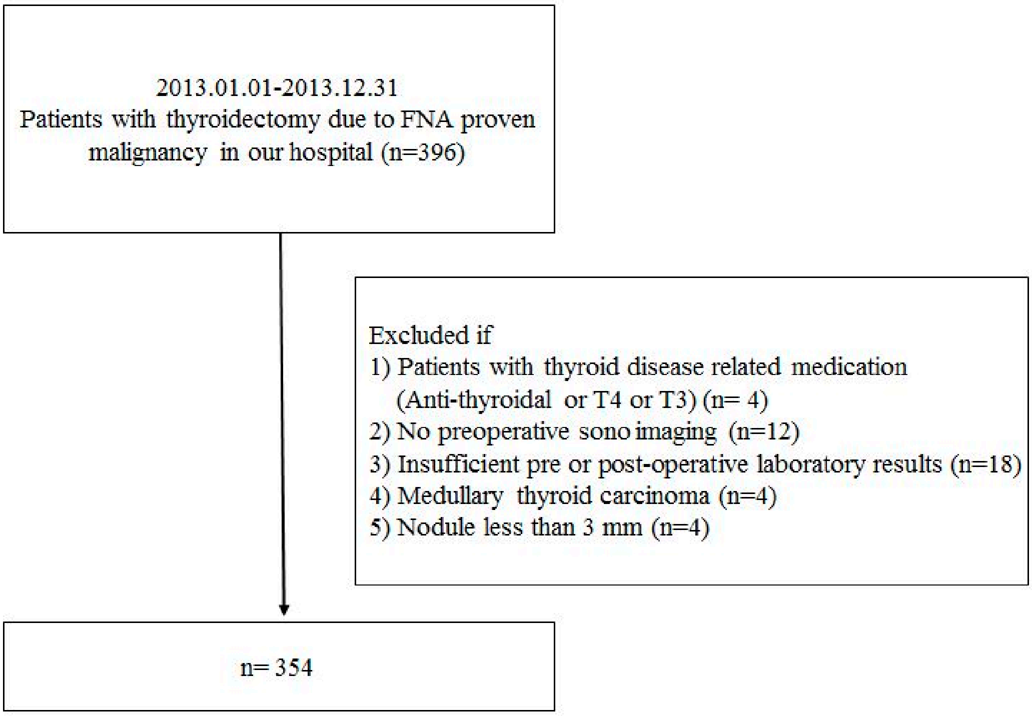

(**b**)

Figure S1. Flow chart for study population enrollment. (**A**) Group for observing the association of calcification in PTC and coronary artery calcification (Group 1); (**B**) Group for observing the association of calcification pattern in PTC according to thyroid hormones (Group 2).

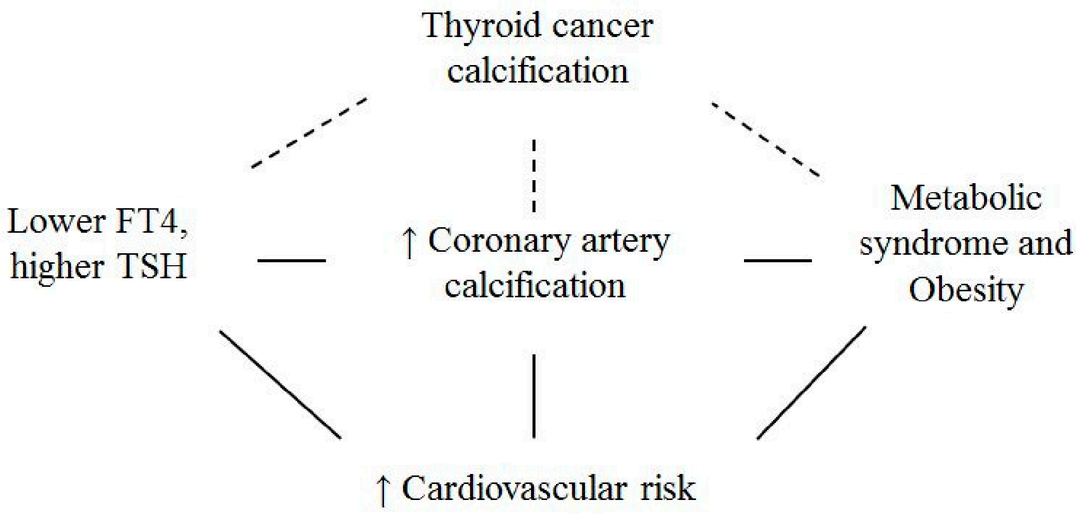

Figure S2. A schematic diagram of the study hypothesis. The solid lines indicate knowledge confirmed by several references, and the dotted lines indicate the hypothesis tested in this study. The mechanism for the relationships has not been clarified, even for information confirmed by published references.
